# Effective Therapeutic Options for Melioidosis: Antibiotics versus Phage Therapy

**DOI:** 10.3390/pathogens12010011

**Published:** 2022-12-21

**Authors:** Yue-Min Lim, Jamuna Vadivelu, Vanitha Mariappan, Gopinath Venkatraman, Kumutha Malar Vellasamy

**Affiliations:** 1 Department of Medical Microbiology, Faculty of Medicine, Universiti Malaya, Kuala Lumpur 50603, Malaysia; 2 Centre of Toxicology and Health Risk Studies (CORE), Faculty of Health Sciences, Universiti Kebangsaan Malaysia, Kuala Lumpur 50300, Malaysia; 3 Universiti Malaya Centre for Proteomics Research, Universiti Malaya, Kuala Lumpur 50603, Malaysia; 4 Department of Biochemistry, Saveetha Dental College, Saveetha Institute of Medical & Technical Sciences, Saveetha University, Chennai 600 077, India

**Keywords:** melioidosis, *Burkholderia pseudomallei*, phage therapy, antibiotics, antibiotics resistance

## Abstract

Melioidosis, also known as Whitmore’s disease, is a potentially fatal infection caused by the Gram-negative bacteria *Burkholderia pseudomallei* with a mortality rate of 10–50%. The condition is a “glanders-like” illness prevalent in Southeast Asian and Northern Australian regions and can affect humans, animals, and sometimes plants. Melioidosis received the epithet “the great mimicker” owing to its vast spectrum of non-specific clinical manifestations, such as localised abscesses, septicaemia, pneumonia, septic arthritis, osteomyelitis, and encephalomyelitis, which often lead to misdiagnosis and ineffective treatment. To date, antibiotics remain the backbone of melioidosis treatment, which includes intravenous therapy with ceftazidime or meropenem, followed by oral therapy with TMP-SMX or amoxicillin/clavulanic acid and supported by adjunctive treatment. However, bacteria have developed resistance to a series of antibiotics, including clinically significant ones, during treatment. Therefore, phage therapy has gained unprecedented interest and has been proposed as an alternative treatment. Although no effective phage therapy has been published, the findings of experimental phage therapies suggest that the concept could be feasible. This article reviews the benefits and limitations of antibiotics and phage therapy in terms of established regimens, bacterial resistance, host specificity, and biofilm degradation.

## 1. Introduction

In 1911, Captain Alfred Whitmore and his aide C. S. Krishnaswami observed a “glanders-like” illness among morphine addicts in Rangoon General Hospital, Burma, and named it Whitmore’s disease in his honour [1]. The condition was then renamed melioidosis by Ambrose T. Stanton and William Fletcher due to its resemblance to the distemper of asses [2]. The researchers identified *Burkholderia pseudomallei* (previously known as *Pseudomonas pseudomallei*) as the causative bacterium [2]. *B. pseudomallei* is a small, rod-shaped, aerobic, Gram-negative, bipolar, non-spore-forming, and motile saprophyte that can affect humans, animals, and sometimes plants. It is found in contaminated wet soil and water in natural habitats, including ponds, lakes, rivers, and seas. The Centers for Disease Control and Prevention (CDC) has included *B. pseudomallei* in Tier 1 select agent list on account of its high resulting fatality rate, antibiotic resistance, widespread distribution in endemic regions, and the risk of aerosol spread [3,4].

Today, melioidosis remains a potentially fatal, difficult-to-manage infection, with a 10–50% mortality rate [5]. The disease is prevalent in tropical and subtropical areas, particularly in Southeast Asia and Northern Australia [3,6]. However, it was believed to have a wider global distribution as the infection had the potential to spread epidemically to non-endemic regions [7]. According to estimates, melioidosis was underreported in 45 countries and was likely to be present in 34 countries [8]. Annually 165,000 cases of human melioidosis were estimated to occur worldwide, with approximately 54% of these cases resulting in deaths [8]. 

Melioidosis received the epithet “the great mimicker” owing to its vast spectrum of non-specific clinical manifestations, such as localised abscesses, septicaemia, pneumonia, septic arthritis, osteomyelitis, and encephalomyelitis, which often lead to misdiagnosis and ineffective treatment [9]. Currently, antibiotics are the most effective therapeutic option, which includes intravenous therapy with ceftazidime/meropenem, followed by oral therapy with trimethoprim-sulfamethoxazole (TMP-SMX; or co-trimoxazole) or amoxicillin/clavulanic acid (or co-amoxiclav) and supported by adjunctive treatment. However, *B. pseudomallei* is intrinsically resistant to several antimicrobial agents, which restricts the treatment options available for melioidosis [10]. Although uncommon, *B. pseudomallei* also developed acquired resistance to clinically significant drugs, resulting in treatment failure and substantial mortality rates [11]. Therefore, phage therapy has been proposed as an alternative treatment. This article reviews the current advancements in melioidosis treatments, including their benefits and limitations in terms of established regimen, bacterial resistance, host specificity, and biofilm degradation.

## 2. Antibiotic Therapy

### 2.1. Introduction to Antibiotics

Antibiotics refer to any compound or agent produced by microorganisms through chemical synthesis that can antagonise other living microorganisms and combat bacterial infection [12]. They work as bactericidal or bacteriostatic, depending on their mode of action. Bactericidal antibiotics kill bacteria by interfering with their cell wall structure, whereas bacteriostatic drugs inhibit bacterial replication by inhibiting DNA replication, protein synthesis, and nutrition delivery. The golden age of antibiotics began in 1929 when Sir Alexander Fleming discovered penicillin, the world’s first and most frequently used antibiotic [13]. Antibiotics have saved millions of lives and marked a great leap forward in modern medicine. Over time, a wide variety of natural, semi-synthetic, and synthetic antibiotics have been developed, and they have become the most common treatment for bacterial infections. Existing antibiotics are also chemically modified to produce new-generation antibiotics that may be more effective against certain diseases [14].

### 2.2. Treatment for Melioidosis

Although melioidosis has been recognised since the 19th century, antibiotics remain the only commercially available treatment due to a dearth of research into alternative therapeutic options [15]. The antibiotic treatment is often challenging because *B. pseudomallei* demonstrates intrinsic resistance to ampicillin, penicillin, streptomycin, gentamicin, tobramycin, as well as first- and second-generation cephalosporins [16]. Additionally, the rapid spread of the disease and the tendency of *B. pseudomallei* to develop latent infections render the treatment prolonged and biphasic [17]. The 2010 HHS *Burkholderia* Workshop concluded the standardised regimen for melioidosis treatment and post-exposure prophylaxis [18]. Briefly, a two-phase antibiotic therapy was recommended: the acute phase, which aims to treat sepsis upon confirmed diagnosis, and the eradication phase, which aims to destroy any remaining bacteria and reduce the risk of recrudescence and relapse.

#### 2.2.1. Acute Phase

The acute phase, also known as the initial intensive phase, is the first-line treatment for acute melioidosis via intravenous administration of high-dose antibiotics in order to prevent mortality [19]. Current treatment consists of a minimum 14-day course of either a cephalosporin, often ceftazidime, or a carbapenem, typically meropenem (Table 1). Among them, ceftazidime is commonly prescribed, while meropenem is reserved for patients with life-threatening infections who must be admitted to the Intensive Care Unit (ICU) [20,21]. In situations of privileged site infection, such as central nervous system (CNS) infection, septic arthritis or osteomyelitis, cutaneous melioidosis, and deep-seated or organ abscesses, TMP-SMX is recommended for use in the treatment [21,22]. Consequently, such dual therapy can enhance tissue penetration, minimise the risk of developing resistance, and has no impact on mortality rates [23].

In an attempt to determine the optimal antibiotic regimen, several clinical studies in Thailand and Australia assessed the effectiveness and safety of the drugs. Since the discovery of melioidosis, conventional therapy using chloramphenicol, doxycycline, and TMP-SMX had been adopted [24]. However, the regimen was discontinued in 1985 due to a high mortality rate of 37.9 to 61%, with septicaemia and multiple foci accounting for 87% of deaths [24]. To address this issue, ceftazidime, which had been proven active in vitro and had a more promising effect for eradicating *B. pseudomallei,* was added to the treatment regimen [25,26]. Comparing ceftazidime treatment to conventional therapy in a randomised trial with 161 culture-positive patients, the overall mortality rate decreased by 50% (37% vs. 74%; 95% confidence interval 19–81%) [27]. The outcomes favoured ceftazidime for treating severe melioidosis. 

Subsequently, in vitro activity studies revealed that carbapenems were more effective against *B. pseudomallei* than ceftazidime or co-amoxiclav [28]. Theoretically, carbapenems were also preferred over ceftazidime because of their extended duration of post-antibiotic effect and lower endotoxin release [29,30]. In a six-year retrospective analysis of severe melioidosis treatment in Darwin, both meropenem-treated (63 patients) and ceftazidime-treated (153 patients) groups achieved comparable results, with a 19% mortality rate, despite an intentional selection bias against more-ill patients to receive meropenem [20]. Among carbapenems, meropenem was favoured over imipenem as a therapeutic option due to its lower seizure risk and more convenient dosing schedule [20].

Over two decades, the Darwin melioidosis guideline administered under the Darwin Prospective Melioidosis Study (DPMS) progressively refined the approach to melioidosis diagnosis and therapy, with over 1150 culture-confirmed cases at Royal Darwin Hospital (RDH) [23,31]. The 2020 revision of the 2015 guideline extended intravenous antibiotic treatment during the acute phase beyond the recommended two weeks, resulting in a low rate of mortality, relapse, and recrudescence [22,23]. Based on the clinical experience of RDH clinicians, the acute phase treatment has been prolonged to a minimum of three weeks to cure concurrent bacteraemia and pneumonia with only a single lobe, as well as unilateral and bilateral multi-lobar pneumonia without bacteraemia (Table 1). In addition, patients with concurrent bacteraemia and unilateral or bilateral multi-lobar pneumonia are treated for a minimum of four weeks. However, this guideline is used as the standard for treatment recommendations in the Australian region. Other countries administer different or modified therapeutic guidelines based on their national standards and policies.

#### 2.2.2. Eradication Phase

As the acute-phase treatment continued to evolve and improve, the number of patients surviving infection during the initial phase increased. However, DPMS conducted in the Northern Territory of Australia over 23 years (1989–2012) showed that among 679 patients who survived initial melioidosis, 39 (5.7%) had a recurrence, 29 (4.3%) had a relapse, and 10 (1.5%) experienced re-infection with a different strain of *B. pseudomallei* [32]. Additionally, an estimated 22,500 American soldiers who acquired melioidosis in Vietnam experienced a severe recurrence in later life, earning melioidosis the moniker "Vietnamese time bomb" in the United States [33]. In order to fully eliminate any residual infection that could relapse, eradication therapy is recommended as an extended duration after acute-phase therapy [34]. Notably, the initial intravenous and oral eradication therapies need to overlap, as this is when certain patients are at high risk of septicaemic relapse [34,35]. 

Oral eradication therapy, previously known as maintenance therapy, involves oral administration of TMP-SMX (or co-trimoxazole) for a minimum of 12 weeks (Table 1). In cases of sulphonamide allergy, TMP-SMX intolerance, or in pregnant women and children, amoxicillin-clavulanic acid is served as a second-line drug [36]. The duration will be prolonged to 24 weeks for patients with arterial infection, CNS infection, and osteomyelitis [22]. The treatment duration and the agent employed in eradication therapy are two of the most important risk factors associated with relapse [37]. Due to its subclinical and latent nature, *B. pseudomallei* can escape autophagosomes in host phagocytic cells, evade autophagy, and thus avoid host immune response mechanisms of clearance [38,39,40]. Consequently, patients who do not receive long-term eradication therapy are exposed to a significant risk of relapse and acquiring severe melioidosis, with mortality rates comparable to those who do not receive treatment [18]. It is evident from an open randomised study that the 20-week therapy had a 59% lower relapse rate than the 8-week therapy (10% vs. 23%) [35,41]. 

Numerous studies were conducted to determine the drug of choice and thus enhance therapeutic efficacy. In the first open randomised trial in Ubon Ratchathani between 1989 and 1992, the conventional regimen of chloramphenicol, doxycycline, and TMP-SMX was compared to the co-amoxiclav regimen for a 20-week full eradication therapy [41]. The findings showed that the conventional regimen was associated with a lower relapse rate than the co-amoxiclav regimen (4% vs. 16%), implying that co-amoxiclav could be less effective [41]. In addition, the conventional regimen was estimated to be 15 times cheaper than co-amoxiclav. However, co-amoxiclav was deemed safer and recommended for children, pregnant women, and nursing mothers. This is because antibiotics in conventional regimens antagonise each other in vitro; for instance, trimethoprim (TMP) or sulfamethoxazole (SMX) suppressed the bacteriostatic action of doxycycline and chloramphenicol [42,43]. The study by Dance (1989) revealed that certain *B. pseudomallei* strains tested were highly resistant to chloramphenicol and often exhibited cross-resistance to TMP and SMX [44].

Since conventional antibiotics demonstrated in vitro mutual antagonism and triggered potentially adverse side effects, multiple trials were performed to evaluate the drugs that could be excluded without compromising treatment. The study comparing doxycycline monotherapy to the conventional regimen found that the doxycycline alone regimen had higher relapse and treatment failure rates (25.6% and 46.5%, respectively) than the conventional regimen (2.27% and 18.2%, respectively), leading the researchers to conclude that doxycycline monotherapy was insufficient to be prescribed as a first-line eradication therapy [45]. The succeeding study compared the conventional four-drug regimen to the three-drug regimen comprising TMP, SMX, and doxycycline to assess the impact of omitting chloramphenicol, whose toxicity might be the source of adverse side effects, especially anaemia [46]. According to the findings, the three-drug regimen demonstrated a lower relapse rate of 5.6% and a decreased patient treatment switching rate of 19%, indicating high efficacy and better tolerance and suggesting that chloramphenicol could be omitted [46]. Later, the Melioidosis ERadication THerapy (MERTH) study compared the efficacy and adverse effects of the TMP-SMX monotherapy recommended in Australia to the three-drug regimen prescribed in Thailand [47]. The outcomes were similar in terms of recurrence rates of clinically-suspected (3% in both regimens) or culture-confirmed (5% vs. 7%) cases and melioidosis-related (3% vs. 1%) or overall mortality (6% vs. 8%) rates [47]. Additionally, severe side effects were less frequent in patients receiving TMP-SMX monotherapy (39%) compared to those receiving the three-drug regimen (53%) [47]. The findings were supported by a decade-long retrospective review [48]. In summary, TMP-SMX monotherapy had supplanted the three-drug regimen as the treatment of choice due to its comparable efficacy and fewer adverse effects.

#### 2.2.3. Adjunctive Treatment

Adjunctive treatment, also called supportive treatment, is a form of patient management aimed to minimise in-hospital mortality in patients suffering from severe melioidosis and septicaemia, which were more prevalent among the elderly in Malaysia [49]. Severe melioidosis might be accompanied by complications such as acute respiratory distress syndrome (ARDS), septic shock, and acute renal failure [50,51]. As many severe melioidosis patients died within the first 48 h of treatment, when antibiotics had little or no effect, several strategies for interfering with the systemic inflammatory disorder and pathogenesis that causes deaths or boosting the host defence system had been attempted [19]. To illustrate, supportive treatment such as abscess drainage, blood pressure maintenance, respiratory and acute renal failure management, and appropriate glycaemic control should be administered [19]. Moreover, the patients should be monitored in an ICU or a high-dependency facility [49].

## 3. Antibiotic Resistance Developed by *Burkholderia pseudomallei*

### 3.1. Intrinsic and Acquired Antibiotic Resistance

Over the past few decades, antibiotics’ invention and widespread use triggered the evolution of bacteria with complicated antibiotic resistance (AMR) mechanisms. Infections caused by these antibiotic-resistant bacteria are more difficult to treat and manage than those brought on by non-resistant bacteria. Some bacterial species are intrinsically resistant to an antibiotic without mutation, indicating that the antibiotic cannot ever be used to treat the infections caused by these bacteria. For instance, the intrinsic antibiotic resistance of *B. pseudomallei* limits therapeutic options for melioidosis treatment. *B. pseudomallei* was inherently resistant to first- and second-generation penicillin, streptomycin, aminoglycosides, cephalosporin, polymyxin, quinolones, tobramycin, gentamicin, and macrolide, as well as third-generation penicillin, aminoglycoside, cephalosporin, and rifamycin [7,16]. Thankfully, intrinsic resistance of *B. pseudomallei* to ceftazidime was uncommon and had not been recorded for meropenem [28].

AMR occurs naturally; however, it can be exacerbated by the abuse or unjustified use of antibiotics as prophylactic or therapeutic drugs in animal and human health care or animal husbandry [52]. In 30% to 50% of cases, antibiotic therapy duration, agent choice, or treatment indication was inappropriate [53,54]. Consequently, poorly given antibiotics may promote acquired bacterial resistance to particular antibiotics [55]. Despite its rarity, *B. pseudomallei* was found to acquire resistance to all prescribed drugs, including ceftazidime, meropenem, TMP-SMX, and co-amoxiclav, during treatment [44,56,57,58,59,60,61,62,63].

According to an AMR review in northeast Thailand between 1992 and 2003, 24 of 4021 patients acquired resistance to ceftazidime (0.2%), co-amoxiclav (0.1%), or both antibiotics (0.3%) [64]. To further support the findings, an in vitro antimicrobial activity study in Brazil showed that a high percentage of twenty *B. pseudomallei* strains studied were resistant to ceftazidime (10%) and co-amoxiclav (30%) [65]. Moreover, resistance rates to TMP-SMX were 2.5% (by *E*-test) in Australia, 13% (by *E*-test) in Thailand, and 16% (by microbroth dilution) in Thailand [66,67,68]. The reduced susceptibility of *B. pseudomallei* to meropenem was reported in 11 melioidosis cases, which was associated with an overexpression of the resistance-nodulation-division (RND) efflux pumps [59].

### 3.2. Antibiotic Resistance Mechanisms

While mobile genetic elements, including integrons, transposons, and plasmids, mediate most AMR mechanisms, *B. pseudomallei* employs chromosomally encoded genes [10,69]. This is evidenced by the discovery of seven drug resistance genes that encoded for Ambler class A, B, and D β-lactamases in the *B. pseudomallei* strain K96243 genome [70]. Practically, the most crucial gene was *blaA*_BPS_, which encoded for BPS-1, an Ambler class A β-lactamase that could hydrolyse most cephalosporins, except ceftazidime [7,71,72]. However, a β-lactamase mutant, BPS-1m, with a single amino acid substitution at residue 167, was found in the ceftazidime-resistant strain [73]. The acquired resistance is linked to the *bla* gene mutations that induce the chromosomal β-lactamase modifications, resulting in three distinctive modes: insensitivity to β-lactamase inhibitor inhibition, chromosomal enzyme derepression, and selective hydrolysis of ceftazidime by β-lactamase [56,74]. 

Aside from molecular strategies, biofilm formation by *B. pseudomallei* is another crucial candidate for AMR mechanisms [75]. Biofilms are immobilised microbial populations encased in an extracellular polymeric substance (EPS) matrix consisting of polysaccharides, DNA, and protein [76]. The viscous and dense EPS matrix blocks antibiotics from penetrating bacterial cells, thus shielding them from therapeutic drug attacks [77,78]. Moreover, biofilms restrict oxygen and nutrient availability to sessile bacterial cells, which slows cell division and metabolism, hence tolerating the effect of antibiotics that target fast-growing cells [79,80,81]. Therefore, substantial doses of antibiotics are required to degrade biofilm, yet the biofilm fails to be eradicated and colonies may regrow after treatment [82,83]. In addition, high antibiotic dosage may result in tissue toxicity [84]. As horizontal gene transfer is encouraged by the proximity of bacterial cells, drug-resistance genes may theoretically be shared more easily among biofilm-forming bacteria [85]. 

**Figure 1:**
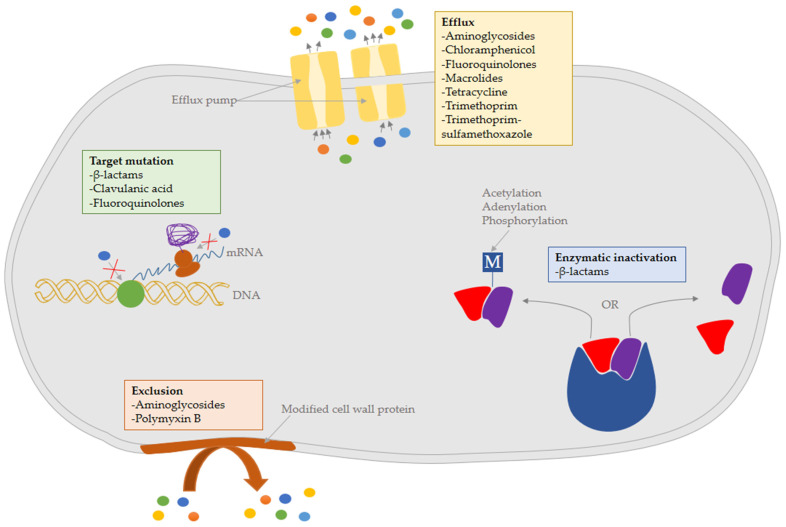
A summary of antibiotic resistance mechanisms in *B. pseudomallei,* including: target site modification; enzymatic inactivation, either by modification or cleavage; efflux from the cell by pumps or transporters; and exclusion from the cell by modified cell wall protein.

The mechanisms of antibiotic resistance in *B. pseudomallei* and their effects on melioidosis treatment had previously been described [10]. Taken together, four main mechanisms, including drug exclusion from the cell due to decreased permeability of the bacterial cell envelope; drug efflux from the cell by pumps or transporters; enzymatic inactivation by alteration or cleavage, and altered target sites, had been discussed (Figure 1). In rare cases, mechanisms such as target overproduction, drug sequestration, and metabolic bypass by replacing a susceptible enzyme or pathway with a resistant variant were also noted.

## 4. Phage Therapy

### 4.1. Introduction to Phage Therapy

Phage therapy (PT) refers to the application of phages to specifically reduce or eradicate pathogenic bacteria in clinical, veterinary, agricultural, and food microbiological fields. Phages, commonly known as bacterial viruses or bacteriophages, are viruses that only infect bacteria and archaea. Phages are composed of three parts: a single type of nucleic acid (either a single- or double-stranded RNA or DNA in a linear or circular configuration), a capsid that protects genetic material from degradation, and the majority have a fibre tail that adsorbs to the host bacterium, facilitates the precise recognition of bacterial-surface-exposed receptors and mediates host attachment [86,87]. Unlike antibiotics, which are made up of chemical compounds, phages are natural bacterial parasites co-evolving with their bacterial hosts due to their rapid replication rates and genome plasticity [88,89]. In the context of PT, only virulent phages capable of directly destructing the pathogenic bacterial cells through the lytic cycle are relevant [89]. 

Owing to the increasing occurrence of antibiotic-resistant bacterial infections, bacteriophages and their potential use in PT have gained tremendous interest [90]. PT is currently making a comeback as an antibiotic-free alternative, but only Georgia and Russia have commercialised it [91,92,93]. Therefore, several initiatives have been formulated and implemented to expedite the development of PT. For instance, the Phage Therapy Unit, the first PT centre to be established in Europe in 2015, has become a global benchmark for other centres addressing the issue of antibiotic resistance [94]. The United State Food and Drug Administration (FDA) regularly permits the compassionate use of PT after all other choices have been exhausted [95].

In recent years, many experimental PTs targeting different pathogenic bacteria, especially multi-drug resistant bacteria, including *Pseudomonas aeruginosa, Acinetobacter baumannii*, and *S. aureus,* have been conducted. A successful 6-phage cocktail therapy, including PAK_P4, PAK_P1, PYO2, E217, E215, and DEV, was shown to reduce *P. aeruginosa* biofilms, cure bacteremia in *Galleria mellonella* larvae, and deal with acute respiratory infection in mice [96]. Furthermore, a novel approach used to prepare personalised therapeutic bacteriophage cocktails was described in order to provide effective therapy for *A. baumannnii* pancreatic pseudocyst infection [97]. In addition, no adverse reactions were found when AB-SA01 was administered intravenously for serious *S. aureus* infections such as septic shock and bacterial endocarditis [98].

### 4.2. Treatment for Melioidosis

According to previous research, *B. pseudomallei* in the environment are regulated by phages in the same ecosystem [99]. Phages are expected to strongly affect the number and pathogenicity of environmental *B. pseudomallei* [100]. Additionally, a high phage titre was found in *B. pseudomallei* containing environmental samples under natural conditions [99,101,102,103]. Therefore, phages are considered viable therapeutic agents, particularly when conventional antibiotics fail to respond. Although no effective phage therapy for melioidosis has been published, the findings of experimental phage therapy suggest that the concept is feasible. 

In this article, we discuss six phages with different *B. pseudomallei* strains (P37, K96243, E0237, CMS, 365A, and HNBP001) as hosts (Table 2) [101,103,104,105,106,107]. Based on their morphological characteristics, four of the phages had contractile tails and were classified into the *Myoviridae* family [103,104,105,106]. Two of the phages had short tails and were classified as the *Podoviridae* family [101,107]. In the cases of staphylophages, *Myoviridae* phages were more common than *Podoviridae* phages in commercial preparations such as therapeutic cocktails [108]. Both *Myoviridae* and *Podoviridae* staphylophages were virulent phages with high lytic capabilities and broad host ranges [108]. The study by Kornieko et al. (2020) also showed that the application of phages with diverse host ranges from different families would boost cocktail efficiency [108]. Future applications of the idea to *B. pseudomallei* phages may yield an effective treatment cocktail. 

All phages demonstrated broad strain infectivity ranges (53.5–100%), including antibiotic-resistance strains [106], and were considered host-specific since they only infected *B. pseudomallei* and the closely-related species, *B. mallei* and *B. thailandensis*. Introducing cocktails containing modified derivatives from repeated propagation in phage-resistant strains could further boost lytic capabilities [109]. Some phages were able to kill both biofilm-forming and non-biofilm-forming *B. pseudomallei* [106,109]. The efficiency of *B. pseudomallei* phages has been evaluated in vitro with A549 cells and in vivo with BALB/c mice and *C. elegans*, resulting in the rescue of infected organisms and reduction in bacterial burden [105,107]. The therapeutic efficiency can be further increased by introducing a combination therapy of phages and antibiotics [107].

#### 4.2.1. Phages ST79 and ST96

In 2011, Yordpratum and colleagues reported the first comprehensive study of *B. pseudomallei*-lysing phages isolated from soil [103]. Six *Myoviridae* lytic phages capable of lysing clinical *B. pseudomallei* strain P37, designated ST2, ST7, ST70, ST79, ST88, and ST96, were isolated from soil samples collected in Nampong District, Khon Kaen Province, Thailand. They had double-stranded DNA genomes between 24.0 and 54.6 kb in size. Moreover, these phages infected a wide range of *B. pseudomallei* (41–78%) and were host-specific, except that all phages could produce small plaques on *B. mallei* and phages ST2 and ST96 were able to infect *B. thailandensis*. Among six phages, phages ST79 and ST96 could achieve the highest titre at an optimal multiplicity of infection (MOI) of 0.1 within six hours. Then, phage ST79, with a broader strain infectivity range of 71%, was chosen to characterise the growth parameter further. As determined by the one-step growth curve [110], the eclipse, latent times, and burst size of phage ST79 were 20 min, 30 min, and 304 PFU/infected cells, respectively. 

In a more recent study using the microplate phage virulence assay [111], all six phages were proven to be more effective at lysing *B. pseudomallei* soil strains compared to the clinical strains, with phage ST79 exhibiting the highest lytic capability at 61% [109]. A cocktail of six phages was found to have a similar lytic capability (62%) as individual phages, suggesting that all phages possessed the same lytic activities. The lytic capability of the cocktail was boosted to 80% by adding three ST79 derivatives modified through repeated propagation in phage-resistant strains. Regardless of MOI, phage ST79 reduced the bacterial number by 4-log after four hours of treatment, but the bacteria grew back after 12 h. To combat this, the 6-phage cocktail was suggested due to its ability to suppress bacterial regrowth for at least 24 h. Furthermore, colorimetric analysis proved that phage treatment at an MOI of 10 notably reduced the biofilm formation by high (strain H777), moderate (strain 844), and low (strain H1038) biofilm forming *B. pseudomallei*. The efficiency varied according to the amount of biofilm formed by different strains and the stage of biofilm formation prior to phage treatment. Overall, these phages could serve as biocontrol agents for environmental *B. pseudomallei* or alternative therapies for human melioidosis.

#### 4.2.2. Phage ΦBp-AMP1

A study in Khon Kaen Province, Thailand, discovered ΦBp-AMP1, the first environmentally isolated podovirus effective against *B. pseudomallei*, using Thai clinical isolate K96243 as host [101]. It was a member of the *Podoviridae* family based on its 45-nm-wide icosahedral capsid and 20-nm-long, typically short podovirus tail. The genome of the phage is 45 Kb in size. ΦBp-AMP1 was highly host-specific, as it could not infect *E. coli*, *P. aeruginosa*, *B. vietnamiensis*, *B. ubonensis*, *B. multivorans*, *B. thailandensis*, and *B. cepacia.* Also, the phage exhibited a broader range of *B. pseudomallei* infectivity, lysing all 11 clinical strains studied, though it was more effective against Thai strains than Australian strains. 

Using *B. pseudomallei* K96243 as its host, the one-step growth curve [110] for ΦBp-AMP1 exhibited an eclipse time of 40 min, a latent time of 60 min, and a burst size of 158 ± 54. Furthermore, the thermal stability test revealed that the phage was stable for at least eight hours at 50 °C but lost viability at 60 °C, as was typical for phages. According to molecular analysis, ΦBp-AMP1 carried a gene homologous to the open reading frame (ORF) that encoded tail tubular protein B (TTPB) in a temperate *B. thailandensis* phage, which served as a phylogenetic marker in prior research. Then, the phylogenetic tree at the amino acid level suggested that ΦBp-AMP1 was distantly related to known phages but closely connected to the T7-like *Ralstonia* phage RSB1 and the *B. thailandensis* MSMB43 prophage. In summary, ΦBp-AMP1, the first *B. pseudomallei* podovirus, could demonstrate antimicrobial capabilities distinct from myoviruses, making it a novel possibility for *B. pseudomallei* treatment.

#### 4.2.3. Phage φX216

In a study by Kvitko et al. (2012), the Thai environmental strain E0237 of *B. pseudomallei* had been reported to spontaneously release a phage named φX216 [104]. One-step growth curve experiments [112] indicated that the latent phase, life cycle, and burst size of φX216 were 60 min, 80 min, and 120 PFU/infected cells, respectively. Compared to the previous study, φX216 exhibited one of the widest ranges of strain infectivity at 77.8%. Although φX216 lysed all nine *B. mallei* strains studied, it was deemed highly host-specific because it did not infect closely related or other *Burkholderia* species. Experiments with *B. mallei* strains had proven that lipopolysaccharide (LPS) O-antigen might be employed by φX216 as a host receptor, whereas *B. pseudomallei* might employ a different receptor which could be lacking in *B. mallei.*

According to the sequencing results, φX216 was identified as a P2-like phage from the family *Myoviridae* subgroup A. The entire genome (GenBank: JX681814) was 37,637 bp long and was predicted to contain 47 ORFs. Predicted regions of the genome were correlated with DNA replication and lysogeny, tail structure and assembly, host cell lysis, and capsid structure. The pairwise alignment between the genomes of φX216 and *B*. *pseudomallei* Pasteur 52237 (GenBank: DQ087285.2) isolates φ52237 showed a high similarity of 99.8%, and these phages were also found to have identical strain host range. 

PCR analysis showed that DNA fragments indicating the presence of P2-like prophages were amplified in 41.7% of the strains studied, demonstrating the prevalence of P2-like prophages among *B. pseudomallei* isolates. Instead, it appeared that φX216 could infect the prophage-carrying strains efficiently since they had been infected by other P2-like phages and converted into lysogens. In conclusion, the high species specificity and wide range of strain infectivity of φX216 demonstrated that it had the potential to be selected as a suitable option for designing rapid phage-based *B. mallei* and *B. pseudomallei* detection assays.

#### 4.2.4. Phage C34

In a previous study in our laboratory in Kuala Lumpur, Malaysia, we had successfully isolated phage C34 from seawater samples obtained in Port Dickson, Negeri Sembilan, using a local clinical *B. pseudomallei* isolate, CMS [105]. Phage C34 was identified as a *Myoviridae* phage with double-stranded DNA, a 50-nm-wide head, and a 138-nm-long contracted tail. The phage was exclusively host-specific, infecting 53.5% of *B. pseudomallei* clinical isolates in our collection but not *B. thailandensis*, *B. cepacia*, and *P. aeruginosa.* The phage reduced the number of bacteria by 4-log units in the first hour of treatment at an optimal MOI of 10. The one-step growth curve [113] showed that phage C34 had an eclipse time of 30 min, a latent time of 40 min, and a burst size of 234 PFU/infected cells. Furthermore, seven out of 15 mucoid colonies of *B. pseudomallei* were identified as bacteriophage insensitive mutants (BIMs), with six growing more slowly than the wild-type strain.

To study phage efficacy in prophylaxis, approximately 2 × 10^5^ human lung epithelial cells, A549, were pre-treated overnight with 2 × 10^7^ PFU of phage C34 before being infected with 2 × 10^6^ CFU of *B. pseudomallei*, resulting in a 20% increase in cell survivability from 22.8 ± 6.0% (non-pre-infection treated control) to 41.6 ± 6.8%. However, infected cells treated with phage particles before and after infection showed no improvement in survivability due to *B. pseudomallei* infection limiting phage C34 internalisation or permeabilisation into A549 cells, hence diminishing the efficacy of post-infection treatment. In addition, the survivability of A549 cells was not affected by the phage application (up to 2 × 10^7^ PFU), as shown by no discrepancies in the survivability of uninfected cells and uninfected cells treated with phage.

BALB/c mice (*n* = 15 per group) infected intranasally with 100 CFU of *B. pseudomallei* were used to elucidate the therapeutic and antimicrobial activity of phage C34 in an in vivo model. In comparison to untreated controls (*p* < 0.001), intraperitoneal administration of phage (2 × 10^8^ PFU) 24 h before and 2 h after exposure to bacteria (*p* = 0.7006 in both groups) substantially protected infected mice. At the end of the experiment, five mice from both pre-and post-infection treatment groups survived, demonstrating the successful rescue of 33.3% of *B. pseudomallei*-infected mice. Additionally, BALB/c mice (*n* = 18 per group) treated post-infection had a lower average bacterial burden in the spleen (*p* < 0.01) compared to untreated control, while pre-infection treatment resulted in no significant reduction (*p* > 0.05). The findings strongly implied the potential of phage C34 to be developed as a potent therapeutic agent to cure melioidosis.

#### 4.2.5. Bacteriophage 365A

In a recent study in Thailand, bacteriophage 365A was discovered spontaneously during bacteriophage screening using ceftazidime-resistant *B. pseudomallei* 365A [106]. Bacteriophage 365A was classified as a member of the *Myoviridae* family based on its structure: a 50-nm-wide icosahedral head, a 148-nm-long contractile tail with tail fibres, and double-stranded DNA. The whole-genome sequencing showed a genome size of around 28 kb. Bacteriophage 365A exhibited high strain infectivity by lysing 77% of ceftazidime-susceptible *B. pseudomallei* and all five ceftazidime-resistant strains (365A, 316C, 979B, EPMN34, and EPMN159). It could also form clear zones on closely related *B. pseudomallei* species, including 40% of *B. thailandensis* and 60% of *B. mallei* isolates. However, other Gram-positive and Gram-negative bacteria, such as *B. cepacia*, *P. aeruginosa*, *E. coli*, *A. baumannnii*, *S. aureus*, *Bacillus cereus*, and *Listeria monocytogenes*, could not be lysed.

Bacteriophage 365A exhibited similar antimicrobial activity against ceftazidime-susceptible *B. pseudomallei* strain P37 in the planktonic form at MOIs of 0.1, 1.0, and 10. After 4 h of phage exposure, the viable bacterial counts of *B. pseudomallei* ceftazidime-resistant strains 979B and 316C, and planktonic strain P37 dropped by roughly 3- and 2-log units, respectively. However, by 6 h, all three bacterial strains began to regrow, and until 24 h, the viable counts were no longer significantly different from the control. Furthermore, these three *B. pseudomallei* strains in biofilm conditions were used to study the biofilm reduction ability of bacteriophage 365A at different MOIs. As a result, bacteriophage 365A at MOI of 10 considerably reduced biofilm formation by 60%, 68%, and 80% in *B. pseudomallei* ceftazidime-resistant strains 979B and 316C, as well as planktonic strain P37, respectively, in comparison to the control group (*p* < 0.05). Overall, bacteriophage 365A had proven effective against both planktonic and biofilm forms of ceftazidime-resistant *B. pseudomallei*, despite the observation of bacterial regrowth.

#### 4.2.6. Phage vB_BpP_HN01

Phage vB_BpP_HN01, the first *B. pseudomallei* phage found in Hainan, China, was among over 20 phages isolated from samples obtained near households afflicted by a melioidosis outbreak using the representative Hainan clinical strain HNBP001 [107]. Based on its icosahedral head size of 62.0 ± 1.3 nm and a short tail length of 20.4 ± 0.7 nm, the phage was assigned to the *Podoviridae* family. The phage vB_BpP_HN01 showed high strain infectivity and host specificity, infecting 96% of *B. pseudomallei* strains tested but none of the other bacteria, including *E. coli*, *P. aeruginosa*, *A. baumannii*, and *Klebsiella pneumoniae*. Furthermore, the phage demonstrated favourable thermal and pH stability, surviving at 24–60 °C or pH 3–12 for 30 min. At an optimal MOI of 0.1, phage vB_BpP_HN01 could obtain a high titre of around 10^12^ PFU/mL with an eclipse time of 20 min and a latent time of 40 min. During the one-step growth curve [114], the host population decreased sharply after 30 min of incubation and was fully lysed after 150 min. 

For genomic analysis, the double-stranded linear DNA genome of the phage had a length of 71,398 bp and a tRNA-Asn inferred as an infection-associated codon usage bias. There were 93 ORFs in the genome accountable for maintaining structural pattern uniformity and replicating genetic materials. As determined by BLASTn, the genome shared low sequence similarity with *Achromobacter* phages and the result was corroborated by ViPTree analysis. Additionally, the ViPTree analysis of different phylogenetic patterns showed that the phage might be closely related to *Rhizobium* phages, *Erwinia* phages, and the *Pseudomonas* phage Zuri. 

The therapeutic efficacy of the phage vB_BpP_HN01 against *B. pseudomallei* was experimentally evaluated using A549 cells in vitro and *C. elegans* in vivo. As a result, treatment with phage alone outperformed treatment with ceftazidime alone, successfully rescuing 70.6 ± 6.8% (MOI = 0.1) and 85.8 ± 5.7% (MOI = 1) of infected A549 cells. The cell viability reached a new high of 91.9% (MOI = 0.1) and 96.8% (MOI = 1) when the phage was coupled with ceftazidime. In combination therapy, Phage-Antibiotic Synergy (PAS) had been described with different phages and antibiotics, presumably because antibiotics aided in phage production and accelerated phage-induced lysis [115]. In the model of infected *C. elegans* treated with phage, mortality was reduced by 90% and the bacterial burden, especially in the intestine, also declined. Depending on its lytic capability and stability, phage vB_BpP_HN01 could serve as a treatment for melioidosis.

## 5. Antibiotics vs. Phage Therapy

### 5.1. Well-Established Regimen

Antibiotic therapy remains the mainstay of human melioidosis treatment despite the availability of numerous alternatives with varying degrees of success [15]. A series of antibiotic prescribing guidelines with a robust regulatory approval framework have been documented and implemented with minor modifications according to the policies and resources of each country [18,22,23]. Antibiotics are irreplaceable since they are readily available and reasonably priced with a prescription from a healthcare professional. In order to maximise therapeutic efficacy and reduce relapse and recrudescence rates, the choice of agent and treatment duration are routinely reviewed and ameliorated [37]. To date, a well-established antibiotic regimen with both acute and eradication phases has been developed. The acute phase, which aims to treat sepsis upon confirmed diagnosis, consists of ceftazidime or meropenem, administered intravenously over a 10- to 14-day period. Then, TMP-SMX or amoxicillin-clavulanic acid is used in the oral eradication phase for three to six months to destroy any remaining bacteria and reduce the risk of recrudescence and relapse. However, the relapse rate remained at 10% even when eradication therapy was extended to 20 weeks [116]. The complete treatment involving first-line agents (ceftazidime + TMP-SMX) also resulted in a 14% mortality rate [117]. 

Since the availability of antibiotics in the 1940s, interest in PT research had waned, but this practice has resurged as an alternative in light of the widespread antibiotic-resistant bacteria [95]. New phages against *B. pseudomallei* can be discovered in days or weeks as they are easily isolated from the environment, especially water and soil. Several phages were found to be effective against *B. pseudomallei*, including ST79 and ST96 [103], ΦBp-AMP1 [101], φX216 [104], C34 [105], 365A [106], and vB_BpP_HN01 [107]. However, PT for melioidosis is currently being reviewed in clinical trials and has not yet been licensed for public use. There are a variety of considerations, such as selection of phage, route of administration, treatment duration, bacterial resistance, and other potential shortcomings associated, which warrants further investigation.

### 5.2. Bacterial Resistance

Owing to its intrinsic resistance to many antibiotics, the antibiotic susceptibility of *B. pseudomallei* is a crucial criterion in determining the best treatment for melioidosis. The study of antibiotic susceptibility patterns of *B. pseudomallei* strains revealed that all clinical isolates tested were completely (100%) susceptible to ceftazidime, amoxicillin-clavulanic acid, imipenem, TMP-SMX, piperacillin-tazobactam, and tetracycline [118]. However, *B. pseudomallei* has been reported to develop acquired antibiotic resistance in vivo during treatment, which can be fatal if treatment is not shifted to alternative drugs in due course [119]. Considering that ceftazidime is commonly used as a first-line agent in the acute phase, resistance had been observed during treatments [56,64,120]. This was often conferred by mutations in the *penA* gene encoding class A β-lactamase, resulting in altered substrate specificity [60,121]. Resistance to amoxicillin/clavulanic acid (30%) and ceftazidime (10%) was also prevalent among *B. pseudomallei* strains in Brazil [65]. In addition, TMP-SMX resistance emerged during eradication therapy, with rates ranging from 2.5% in Australia to 16% in Thailand [66,67,68]. Favourably, resistance to second-line drugs, including co-amoxiclav, meropenem, and imipenem, was relatively rare [60]. Therefore, second-line treatments are reserved for patients who do not respond to the primary therapeutic options.

In comparison, the phages are host-specific, which limits the infectivity range, minimising the potential of inducing and spreading specific phage-resistant mechanisms [122]. Furthermore, phages are natural bacterial parasites that co-evolve with their bacterial hosts due to their rapid replication rates and genome plasticity [89]. Therefore, phages can spontaneously overcome several bacterial resistance mechanisms, for example, CRISPR-Cas systems, restriction-modification (RM), abortive infection, and adsorption inhibition by developing counter-strategies, including genomic rearrangements, point mutations, production or hijacking of antitoxins, inactivation of the protein involved in bacterial antiphage defence mechanisms (anti-RM and anti-CRISPR systems), and recognition of new or altered bacterial receptors [88,123,124]. For instance, *B. pseudomallei* resistance to phage ST79 was overcome by producing three modified derivatives through repeated propagation in phage-resistant strains, indicating that the resistance could be driven by the RM system [109]. The 6-phage cocktail suppressed the regrowth of *B. pseudomallei* colonies, suggesting that it could inhibit phage-resistant mutants [109]. As observed with phage C34, seven BIMs producing mucoid colonies were identified in A549 cells but lacking in mice due to elimination by the host immune system [105].

### 5.3. Host Specificity

Antibiotics used in melioidosis treatment have proven efficacy against a broad spectrum of Gram-positive and Gram-negative bacteria. According to the 2021 AWaRe Classification Database, ceftazidime and meropenem were in the Watch group, consisting of the most crucial drugs documented in Critically Important Antimicrobials for Human Medicine [125]. TMP-SMX and amoxicillin-clavulanic acid belonged to the Access group since they were active against a diverse set of frequently reported susceptible pathogenic bacteria [125]. The classification indicated that these four drugs were essential for treating not only melioidosis but also other bacterial infections. To illustrate, ceftazidime and meropenem were used to treat *P. aeruginosa* infection [126]; TMP-SMX was the preferred treatment for *Aeromonas* infections and cutaneous nocardiosis [127]; amoxicillin-clavulanic acid was effective against *Streptococcus pneumoniae* [128]. Not only that, these antibiotics were able to treat melioidosis caused by different *B. pseudomallei* strains, as evidenced by the highest susceptibility of the isolates tested to these drugs [119]. However, extensive application of antibiotics in endemic areas accelerated the emergence of acquired bacterial resistance, hence compromising therapeutic efficacy [10]. In addition, these broad-spectrum antibiotics have been shown to induce disruption of the gut microbiota and other common adverse drug reactions. For example, 2% of patients receiving TMP-SMX and 6.5% of patients given co-trimoxazole reported gastrointestinal side effects, but at a lower rate than those taking the conventional regimen [47,48]. Adverse effects, including gastrointestinal disorders, bone marrow suppression, acute kidney injury, and drug reaction with eosinophilia and systemic symptoms (DRESS), were reported in 30% of TMP-SMX treated patients, requiring a therapy switch, cessation, or dosage reduction [129].

Unlike antibiotics, phages are host-specific, infecting only a single species or a few strains within a species of host bacteria. As proof, *B. pseudomallei* phages could not infect other Gram-positive and Gram-negative bacteria, such as *P. aeruginosa*, *B. cepacia*, *E. coli*, *B. vietnamiensis*, *B. ubonensis*, *B. multivorans*, *A. baumannii*, *K. pneumonia*, *S. aureus*, *B. cereus*, and *L. monocytogenes.* Notably, some of them could lyse the host-restricted pathogen *B. mallei* and the non-pathogenic saprophyte *B. thailandensis* [130]. This was not surprising given the high genetic similarity among *B. pseudomallei*, *B. mallei*, and *B. thailandensis* [131,132]. Host-specificity is critical in reducing the risk of phage resistance while minimising the destruction of normal flora bacteria and host cells [122]. However, host specificity impedes therapeutic research and development due to the limited bacterial species or strains that can be infected [133]. Plus, the potential for mass production and distribution was restricted, which is a significant advantage of wide-spectrum antibiotics [133]. Therefore, phage cocktails consisting of multiple lytic phages are introduced and have been confirmed to be effective in vitro against the host bacterium; nevertheless, the success of this strategy relies on identifying the causal pathogen [133].

### 5.4. Biofilm Degradation

While antibiotics could effectively treat infections caused by planktonic bacteria, their efficacy was limited in treating melioidosis, a biofilm-associated bacterial infection [77]. The biofilm matrix produced by *B. pseudomallei* contains microcolonies that are irreversibly adhered to a surface and enclosed in an EPS matrix, restricting drug uptake, and shielding the bacteria from attacks by the host immune system and the antibiotics [85]. Moreover, biofilms contain sessile bacterial cells with slow cell division and metabolism; thus, antibiotics that target fast-growing cells have little or no effect [79]. As horizontal gene transfer is encouraged by the proximity of the bacterial cells, it is theoretically easier to share antibiotic-resistance genes in the biofilm-producing bacteria [85]. This is evident by studies showing that biofilm-associated *B. pseudomallei* demonstrated significantly lower susceptibility and increased resistance to clinically relevant antibiotics compared to those that did not form the biofilm [65,134,135,136]. Furthermore, the high relapse rate of melioidosis was correlated with the biofilm production by the primary infecting isolates but not with the LPS type of *B. pseudomallei* [137]. This could be attributed to the recurrence of biofilm-forming bacteria, which induce antibiotic resistance [135]. In order to penetrate the sticky and thick EPS matrix, antibiotics need to be used in high doses, yet the biofilm fails to be eradicated, and colonies may regrow after treatments [82,83]. Plus, a high dosage of antibiotics may result in tissue toxicity [84].

Phages, especially lytic phages, are not only effective against planktonic *B. pseudomallei*, but they can also prevent and eradicate bacterial biofilms. The ability of phages to reduce biofilm formation is dependent on bacterial cell susceptibility to the phage and the presence of receptors for infection [109]. If the phage is equipped with EPS depolymerase outside the capsid, the biofilm can be quickly dispersed, permitting the phage to penetrate the EPS-enclosed bacteria [133]. In addition, the phage can access the biofilm-forming bacteria via pores or water channels in most biofilms [138]. A biofilm inhibition study at James Cook University in Australia adopted a bacteriocin-like compound produced by *B. ubonesis* and a phage cocktail composed of previously isolated bacteriophages [139]. By 24 h, the levels of inhibition in both treatment groups were comparable (*p* = 0.261); however, by 48 h, only the phage cocktail treatment group demonstrated efficient biofilm inhibition with considerably lower OD than at 24 h due to phage self-replication activity. Six lytic phages, ST2, ST7, ST70, ST79, ST88, and ST96, at MOI of 10, markedly reduced biofilm formation (*p* < 0.05) by *B. pseudomallei* P37 with limited biofilm-forming capacity [109]. In three variants with varying biofilm-forming capacities, including H1038 (low), 844 (medium), and H777 (high), phage ST79 at MOI of 10 could reduce biofilm formation (74.52–95.34%) if treated immediately, (50.21–86.74%) after 3 h attachment, and (27.78–80.75%) after 24 h attachment [109]. Moreover, bacteriophage 365A at MOI of 10 could considerably reduce biofilm formation by 60%, 68%, and 80% in *B. pseudomallei* ceftazidime-resistant strains 979B and 316C, as well as planktonic strain P37, respectively, in comparison to the control group (*p* < 0.05) [106].

## 6. Conclusions

Over the years, melioidosis has remained a challenging condition, with high relapse and mortality rates, and the actual incidence and distribution of melioidosis cases are likely to be considerably underestimated. Currently, there is no authorised licensed vaccine for melioidosis, and the treatment is solely dependent on antibiotics. A well-established antibiotic regimen with explicit guidelines, including acute and eradication phases, and a robust regulatory framework has been developed. However, treatment failure, relapse, and recrudescence have been attributed to the advent of antibiotic-resistant *B. pseudomallei* with biofilm formation and AMR mechanisms mediated by chromosomally-encoded genes. Thus, phages with a narrow host range and low risk of phage resistance have been proposed as an alternative therapeutic agent. In the future, the mortality rate of melioidosis could be dramatically reduced by introducing a quick and easy-to-perform *B. pseudomallei* identification test that allows early diagnosis and treatment of melioidosis. The discovery of novel treatment options, such as new antibiotics, phages, vaccines, and other approaches, can aid in the eradication of *B. pseudomallei* from patients and reduce the risks of bacterial resistance. More focus can be paid to research on rapid diagnostic tests and alternative therapeutics for melioidosis, which will aid in the acquisition of grants and resources.

## Figures and Tables

**Table 1:** A summary of antibiotics treatments for melioidosis.

Application	Drug	Dosage		Route/Frequency	Duration	Remarks
		Adult	Child			
**Acute-phase therapy**						
**Primary therapy** for patients with no complications	Ceftazidime	2 g	50 mg/kg up to 2 g	Intravenous 8-h	Minimum 2 weeks	Minimum duration depends on clinical manifestations ^b^: **2 weeks** (pneumonia without lymphadenopathy or ICU admission, bacteremia with no focus, skin abscess) **3 weeks** (concurrent bacteraemia, pneumonia with only a single lobe, unilateral and bilateral multi-lobar pneumonia without bacteraemia) **4 weeks** (deep-seated infection, concurrent bacteraemia and unilateral or bilateral multi-lobar pneumonia with either ICU admission or lymphadenopathy) **6 weeks** (osteomyelitis) **8 weeks** (central nervous system infection, arterial infection)
**Alternative therapy** for patients who are intolerant to ceftazidime or persistent bacteraemia or with neuromelioidosis	Meropenem	1 g	25 mg/kg up to 1 g	Intravenous 8-h	Minimum 2 weeks	
		2 g ^a^	50 mg/kg up to 2 g ^a^	Intravenous 8-h	Minimum 2 weeks	
**Adjunct therapy** for individuals with serious infections which involves the prostate, brain, or other privileged sites	Trimethoprim/sulfamethoxazole (or co-trimoxazole)	**>60 kg:** two 160 mg/800 mg tablets	8 mg/40 mg per kg; up to 320 mg/1600 mg	Intravenous infusion over 30–60 min 12-h, OR nasogastric, OR oral	Continue for the entire duration after adding it to the treatment	
		**40–60 kg:** three 80 mg/400 mg tablets				
		**<40 kg:** one 160 mg/800 mg tablet OR Two 80 mg/400 mg tablets				
**Eradication-phase therapy**						
**Primary therapy** for patients who are susceptible to co-trimoxazole and do not have a documented allergy to it	Trimethoprim/sulfamethoxazole (or co-trimoxazole)	**>60 kg:** two 160 mg/800 mg tablets	8 mg/40 mg per kg; up to 320 mg/1600 mg	Oral 12-h	12 weeks	Minimum duration depends on clinical manifestations ^b^: **3 months** (skin abscess, bacteremia with no focus, pneumonia with or without either lymphadenopathy or ICU admission, and deep-seated collection) **6 months** (arterial infection, central nervous system infection, osteomyelitis)
		**40–60 kg:** three 80 mg/400 mg tablets				
		**<40 kg:** one 160 mg/800 mg tablet OR Two 80 mg/400 mg tablets				
**Alternative therapy** for patients who are intolerant to co-trimoxazole	Amoxicillin/clavulanic acid (or co-amoxiclav) ^c^	**>60 kg:** three 500 mg/125 mg tablets ^d^	8 mg/40 mg per kg; up to 1000 mg/250 mg	Oral 8-h	12 weeks	
		**<60 kg:** two 500 mg/125 mg tablets ^d^				

^a^ For patients with neuromeliodosis. ^b^ Based on Darwin melioidosis treatment guideline. In the cases of acute-phage therapy, prolongation is determined by the clinical judgment if recovery is sluggish or if blood cultures stay positive after 7 days. ^c^ Co-amoxiclav at a 4:1 ratio is preferable to provide adequate clavulanic acid levels (Cheng et al., 2008). ^d^ Weight-based dosage based on 20 mg/5 mg per kg per dose.

**Table 2:** A summary of phages identified to treat *Burkholderia pseudomallei* infection.

Phage (Host Strain)	Family	Structure	Lytic Capability	Host Range	One-Step Growth	Remarks
**Phage ST79** (P37)	*Myoviridae*	- 54 nm isometric head - 148 nm × 17 nm contractile tail with long tail sheet & tail fibre - 35 kb dsDNA genome (GenBank: NC_021343)	71% (45/63) 61% (61/100) * more effective in lysing soil strains than clinical strains	Can lyse *B. pseudomallei*, and *B. mallei*	- Eclipse period: 20 min - Latent period: 30 min - Burst size: 304 PFU/infected cell	- Three modified ST79 derivatives from repeated propagation in phage-resistant strains could increase lytic capability by 18%. - ST79 & ST96 at MOI of 10 could reduce biofilm formation (*p* < 0.05).
**Phage ST96** (P37)		- 60 nm head - 60 nm tail sheet without tail fibre - estimated 54.6 kb dsDNA genome	67% (42/63) (more effective in lysing soil strains than clinical strains)	Can lyse *B. pseudomallei*, *B. mallei* and *B. thailandensis*	-	
**Phage ΦBp-AMP1** (K96243)	*Podoviridae*	- 45 nm icosahedral capsid - 20 nm podovirus tail - estimated 45 kb genome	100% (11/11) (more effective in lysing Thai strains than Australian strains)	Can lyse *B. pseudomallei* only	- Eclipse period: 40 min - Latent period: 60 min - Burst size: 158 ± 54 PFU/infected cell	- A gene (EMBL: FR850500) identified is homologous to an ORF in a *B. thailandensis* phage that encodes a TTPB. - ΦBp-AMP1 is most strongly connected to the T7-like *Ralstonia* phage RSB1 and the *B. thailandensis* MSMB43 prophage.
**φX216** (E0237)	*Myoviridae* subgroup A	- 37 kb genome (GenBank: JX681814) with 47 predicted ORFs	77.8% (56/72)	Can lyse *B. pseudomallei,* and *B. mallei*	- Latent period: 60 min - Life period: 80 min - Burst size: 120 PFU/infected cell	- 99.8% genomic similarity and identical strain host range with bacteriophage φ52237. - φX216 could infect the prophage-carrying strains efficiently since they had been infected by other P2- like phages and converted into lysogens.
**Phage C34** (CMS)	*Myoviridae*	- 50 nm head - 138 nm contractile tail - dsDNA molecule	53.5% (23/43)	Can lyse *B. pseudomallei* only	- Eclipse period: 30 min - Latent period: 40 min - Burst size: 234 PFU/infected cell	- A549 cells pre-treated with C34 enhanced cell survival by 20%. - C34 treatment resulted in the successful rescue of 33.3% of *B. pseudomallei*- infected mice, as well as a lower average bacterial burden in mice spleen
**Bacteriophage 365A** (365A)	*Myoviridae*	- 50 nm icosahedral head - 148 nm contractile tail with tail fibre - estimated 28 kb dsDNA genome	- 100% (5/5 ceftazidime-resistant strain) - 77% (17/22 ceftazidime-susceptible strain)	Can lyse - 40% (2/5) *B. thailandensis* - 60% (3/5) of *B. mallei*	-	- 365A are effective against ceftazidime-susceptible and ceftazidime-resistant *B. pseudomallei* in planktonic form by 3 log units and 2 log units. - Biofilm formation was reduced by 60–80%
**Phage vB_BpP_HN01** (HNBP001)	*Podoviridae*	- 62 nm icosahedral head - 20.4 nm tail - 71 kb dsDNA genome (GenBank: OM687511) with a tRNA-Asn and 93 ORFs	96% (24/25)	Can lyse *B. pseudomallei* only	- Eclipse period: 20 min - Latent period: 40 min - Total lysis time: 150 min	- High thermal (24–60 °C) and pH (3–12) stability were observed. - The genome shared low sequence similarity with *Achromobacter* phages and other known phages. - With phage alone, 71–86% of infected A549 cells were saved; however 92–97% of cells were rescued when phage was combined with antibiotics. - Infected *C. elegans* treated with phage has lower mortality rate (10%) and bacterial burden.

## Data Availability

Not applicable.
